# Gorham-Stout syndrome presenting in a 5-year-old girl with a successful bisphosphonate therapeutic effect

**DOI:** 10.3892/etm.2012.622

**Published:** 2012-06-26

**Authors:** MIN-WEN ZHENG, MIN YANG, JIAN-XIN QIU, XUE-PING NAN, LU-YU HUANG, WEN-DONG ZHANG, LI GONG, ZHI-ZHONG HUANG

**Affiliations:** 1 Department of Radiation,; 2 Institute of Orthopaedics and Traumatology, Xijing Hospital, The Fourth Military Medical University, Xi’an 710032; Departments of; 3 Urology and; 4 Infection,; 5 The Helmholtz Sino-German Research Laboratory, Department of Pathology,; 6 Department of Information, Tangdu Hospital, The Fourth Military Medical University, Xi’an 710038, P.R. China

**Keywords:** Gorham-Stout syndrome, bisphosphonate, histopathology, radiology, treatment

## Abstract

Gorham-Stout syndrome (GSS), also known as Gorham-Stout disease, massive osteolysis, disappearing bone disease or phantom bone, is a rare disorder of the musculo-skeletal system. It most commonly involves the skull, shoulder and pelvic girdle. Histological examination reveals a progressive osteolysis always associated with an angiomatosis of blood vessels and sometimes of lymphatics, which seemingly is responsible for the destruction of the bone. It is extremely rare that Gorham-Stout syndrome involves the bones of the entire body. A 5-year-old girl complaining of intermittent and dull back pain for 3 months was admitted to a local hospital. X-ray revealed left pleural effusion, and the patient was diagnosed with tuberculous pleurisy. Thus, anti-tuberculosis therapy was performed. However, it was not effective. A soft mass with significant tenderness was found in the upper segment of the right leg 50 days afterwards. X-ray revealed multiple osteolysis of the bilateral clavicle, scapula, rib, vertebral body, ilium, sacrum, femur and tibia. The biopsy from the right tibia disclosed that the lesion was composed of hyperplastic blood vessels and fibrous tissues similar to hemangioma. Based on the above clinical, radiological and histopathological findings, the clinical physician confirmed a diagnosis of Gorham-Stout disease, and prescribed oral anti-osteoclastic medications consisting of bisphosphonates. At present, the girl is alive and healthy, and new lesions have not been noted.

## Introduction

Gorham-Stout syndrome (GSS) is a rare disorder characterized by progressive osteolysis. It was first described in 1838 by Jackson who reported an 18-year-old boy with advanced osteolysis of the humerus (1). In 1955, Gorham and Stout developed histopathological criteria of the disease based on their own experience and the literature findings as follows: ‘Gorhams’s disease is usually associated with an angiomatosis of blood vessels and sometimes of lymphatic vessels, which seemingly are responsible for it’ (2). Currently, approximately 200 cases have been published in the literature to date (3). The majority of cases occur in children and young adults. The clinical presentations are variable and depend on the sites of involvement. GSS most commonly involves the skull, shoulder and pelvic girdle. It rarely involves the bones of the entire body. Thus, we report the case of a girl, 5 years of age presenting with GSS and describe the clinical manifestation, radiological features and histopathological characteristics of GSS and discuss its pathogenetic mechanism, diagnosis and treatment.

## Case report

**Figure 1:**
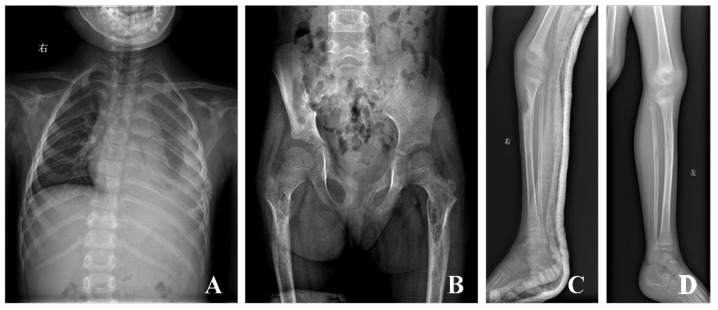
(A) X-ray reveals multiple osteolysis, including bilateral clavicle, bilateral scapula, the 4th, 5th, 9th, 10th right ribs, the 2th and 6th-10th left ribs, the third, fourth and fifth lumbar vertebra, (B) right ilium, sacrum, bilateral femur, (C) right tibia, and (D) left tibia was normal as control.

**Figure 2:**
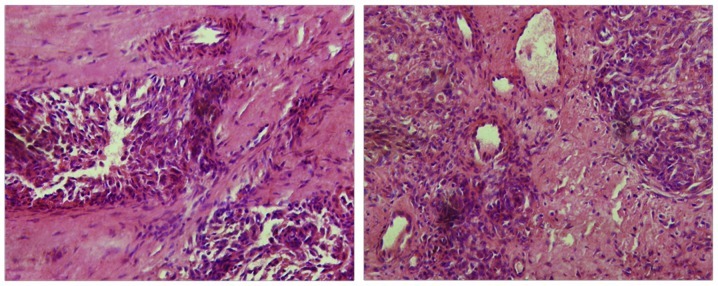
The lesion was composed of hyperplastic blood vessels and fibrous tissues, and the hyperplastic blood vessels were divided into nested structure by fibrous tissues, which was similar to hemangioma.

A 5-year-old girl was referred to a local hospital in 2009 due to back pain with a duration of 3 months. Chest X-ray showed the existence of hydrops in the left thoracic cavity, and tuberculous pleuritis was diagnosed. However, treatment with anti-tuberculosis therapy was ineffective. A soft mass with significant tenderness was noted in the upper segment of right leg 50 days afterward. Thus, the patient presented at our hospital in order to further obtain an accurate diagnosis and receive treatment. X-ray revealed multiple osteolysis of the bilateral clavicle, bilateral scapula, the 4th, 5th, 9th, 10th right ribs, the 2th and 6th-10th left ribs, the third, fourth and fifth lumbar vertebra, right ilium, sacrum, bilateral femur and right tibia (Fig. 1). Laboratory test results included the following: white blood cell count, 8610/*μ*l; hemoglobin, 8.8 g/dl; platelets, 52×10^3^/*μ*l; elevated inorganic phosphorus level (1.98 mmol/l). Other laboratory biochemical and hematological tests, including alkaline phosphatase, lactate dehydrogenase, hydroxybutyrate dehydrogenase and cholesterin were normal. In order to further elucidate the diagnosis, a biopsy from the right tibia was obtained and analyzed. The result disclosed that the lesion was composed of hyperplastic blood vessels and fibrous tissues (Fig. 2), and that the hyperplastic blood vessels were divided into nested structure by fibrous tissues, similar to hemangioma. Moreover, osseous tissues were absent. Based on the above clinical, radiological and histopathological findings, the clinical physician confirmed a diagnosis of Gorham-Stout disease, and prescribed oral anti-osteoclastic medication consisting of bisphosphonates. Three years after the initial operation there was no evidence of new osteolysis.

## Discussion

Gorham-Stout sydrome (GSS) is a rare disorder characterized by progressive osteolysis with invasion of the surrounding soft tissue. It can occur at any age, but is more common in adolescents and young adults (4). There is no racial predilection (4,5). The peak incidence is in the second and third decades of life (5,6) and males are more affected than females (3,6). It may involve different regions of the skeleton, such as the pelvis, clavicle, spine, ribs and facial skeleton. Maxillomandibular lesions are more frequently described when there is associated craniofacial involvement (7).

The exact pathogenetic mechanism of Gorham-Stout syndrome is still unknown. There is controversy even over the presence or absence of osteoclasts in the condition.

Heffez *et al* (5) concluded that osteolysis is due to an increased number of stimulated osteoclasts, and suggested that antiresorptive therapy, for example with bisphosphonates or calcitonin, started in an early phase of the disease could lead to a dramatic improvement in the treatment of progressive osteolytic changes.

The diagnosis of GSS is difficult, particularly in the early stage. It is often misdiagnosed as a neoplasm, pathological fracture, chylothorax or chronic osteomyelitis due to its rarity and bizarre clinical characteristics. GSS occurs in bone and most commonly involves the scapula, clavicle, humerus, thoracic vertebra and rib, and subsequently the lesion gradually involves neighboring bone and surrounding soft tissues. Limb pain, non-shoring physical strength and dysfunction are the main symptoms. X-ray reveals osteolysis. The pathological process is the replacement of normal bone by an aggressively expanding but non-neoplastic vascular tissue, similar to a hemangioma or lymphangioma. Widely proliferating neovascular tissue causes massive bone loss. The clinical presentation consists of pain or pathological fracture. The patient we present is a 5-year-old girl, who presented initially with pain in her back, and subsequently multifocal osteolysis was found. The biopsy revealed that the lesion was composed of hyperplastic blood vessels and fibrous tissues, and the hyperplastic blood vessels were divided into nested structure by fibrous tissues, similar to hemangioma. Moreover, laboratory test results showed that alkaline phosphatase and calcium were normal, whereas inorganic phosphorus was elevated. Thus, hyperparathyroidism caused by the developmental and metabolic disorder of calcium and phosphorus should not be considered. In addition, according to the following diagnostic criteria suggested by Heffez *et al* (5) which include i) a positive biopsy for angiomatous tissue; ii) absence of cellular atypia; iii) minimal or no osteoblastic response and absence of dystrophic calcification; iv) evidence of local progressive osseous resorption; v) nonexpansile, nonulcerative lesion; vi) absence of visceral involvement; vii) an osteolytic radiographic pattern; and viii) negative hereditary, metabolic, neoplastic, immunologic, or infectious etiology, the diagnosis of Gorham-Stout disease was confirmed. Of course, other diseases, including disuse atrophy, acute inflammatory atrophy associated with trauma, primary and metastatic tumors and osteomyelitis, may be excluded based on clinical history, laboratory test results, radiological examination and histopathological findings.

There is no standard therapy available for GSS. A number of different treatments have been proposed, with a huge variation in long-term results (9–12). The prognosis is highly variable and unpredictable, ranging from minimal disability to mortality, depending on the site of involvement, extent of the disease and presence of complications. At present, the treatment for GSS includes radiation therapy, anti-osteoclastic medications (bisphosphonates) and α-2b interferon. Lehmann *et al* (8) reported that long-term bisphosphonate therapy for over 17 years was feasible and could contribute to clinical stabilization in GSS. Moreover, surgical treatment options include resection of the lesion and reconstruction using grafts and/or prostheses. Our patient continued to receive oral anti-osteoclastic medication consisting of bisphosphonates. Three years after the initial operation, there is no evidence of new osteolysis. The patient attends school normally.
